# Enhancing Outcomes in Prosthetic Joint Infections: The Significance of the Periprosthetic Joint Infection Tumor, Node, and Metastasis (PJI-TNM) Classification and Biodegradable Antibiotic Beads

**DOI:** 10.7759/cureus.66012

**Published:** 2024-08-02

**Authors:** Mihnea Popa, Bogdan Cretu, Georgian L Iacobescu, Sergiu Iordache, Adrian Cursaru, Bogdan Serban, Catalin Cirstoiu

**Affiliations:** 1 Orthopedics and Traumatology Department, Bucharest Emergency University Hospital, Bucharest, ROU; 2 Orthopedics and Traumatology Department, Carol Davila University of Medicine and Pharmacy, Bucharest, ROU

**Keywords:** arthroplasty, orthopedic infections, local antibiotic delivery, calcium sulfate beads, pji-tnm classification, periprosthetic joint infections

## Abstract

Background and objectives: Periprosthetic joint infections (PJIs) that occur after hip and knee arthroplasty have a major influence on patient outcomes and healthcare expenses. This study assesses the effectiveness of the PJI tumor, node, and metastasis (PJI-TNM) categorization system and the latest developments in local antibiotic delivery methods for the treatment of PJIs.

Materials and methods: The study involved a retrospective analysis of 23 patients who received treatment for septic hip or knee prostheses at the SUUB Orthopedics and Traumatology Clinic between January 1, 2022, and February 10, 2024. Approval was gained following ethical considerations. Patients were categorized using the PJI-TNM system, and their therapy was customized based on the severity of the infection. The surgical procedures involved either one-stage or two-stage revisions, utilizing vancomycin and gentamicin antibiotic-loaded calcium sulfate beads to administer antibiotics locally. Data pertaining to demographics, clinical characteristics, and microbiology were gathered and examined.

Results: The study comprised 14 male and 9 female patients, with an average age of 68 years. The presence of chronic infections was mostly seen, indicating the development of mature biofilm. Prevalent coexisting medical conditions included diabetes, obesity, and heart failure. The duration of infection control measures was, on average, six months, and 65% of patients reported experiencing enhanced mobility. Acute infections with positive antibiotic responses underwent one-stage modifications. For the majority of patients, a treatment approach involving two-stage modifications, which includes the use of antibiotic-loaded spacers followed by the installation of a prosthesis, proved to be beneficial.

Conclusions: The PJI-TNM classification system improves the management of PJI by offering a systematic method for customized therapy. Calcium sulfate beads, which are biodegradable carriers for antibiotics, provide notable advantages, especially for individuals with severe comorbidities. Continuous progress in diagnostic techniques and localized administration of antibiotics is essential for enhancing the therapy of PJI and improving patient outcomes.

## Introduction

Periprosthetic joint infections (PJIs) following hip and knee arthroplasty are a major problem in orthopedics. They have a severe influence on patient outcomes and also place a considerable financial burden on healthcare systems. Despite the progress made in surgical techniques, preventive measures, and the creation of new materials, the occurrence of PJIs remains unacceptably high, impacting around 0.5-2% of all arthroplasty procedures [1,2]. These infections are concerning because they can greatly reduce the quality of life and cause death rates similar to those of cancer in the first few years after diagnosis [1,3].

In the past, the management of PJIs has been intricate and multifaceted, encompassing a range of approaches from long-term antibiotic therapy to intricate surgical procedures such as debridement and implant retention (DAIR), one-stage and two-stage revisions, and, in severe instances, amputation. Several factors influence the selection of treatment, such as the duration of the infection, the specific bacteria involved, the existence of biofilms, the patient's comorbidities, and the stability of the implant [2,4,5].

The recent implementation of the PJI-TNM categorization system by Baertl et al. is a notable advancement in the effort to establish a consistent approach to assessing and treating PJIs [6]. This method, based on the oncological tumor, node, and metastasis (TNM) classification, takes into account the condition of the infected implant and periarticular soft tissues (T), the specific microorganism causing the infection (N), and host-related variables such as comorbidities (M). The purpose of this classification is to summarize the intricacy of PJIs, offering an organized system to enhance predictions of outcomes and treatment choices when the condition is initially diagnosed. However, the use of this approach in specialized arthroplasty clinics is not widespread, indicating a lack of practical application and the necessity for additional validation to determine its usefulness in clinical settings [2,6,7].

Simultaneously, the pursuit of enhancing local antibiotic administration has resulted in notable advancements in therapeutic approaches. The introduction of antibiotic-loaded, biodegradable carriers such as calcium sulfate beads (e.g., Stimulan rapid cure) represents a significant advancement over conventional polymethylmethacrylate (PMMA) carriers. These more recent polymers provide better antibiotic release patterns and eliminate the requirement for removal, which has been a notable drawback of PMMA beads. These carriers aim to eliminate bacteria that are entrenched in biofilms by achieving high concentrations of antibiotics in the immediate area. This addresses a key problem in the treatment of PJI [8-10].

Moreover, the changing environment of microbial detection, emphasized by the incorporation of bacteriophage-based diagnostics, provides a preview of the next rapid, precise, and sensitive identification techniques that have the potential to completely transform the management of PJIs. These developments highlight the ever-changing nature of PJI management, where continuous research and technical advancements are constantly improving our methods [11-13].

This article seeks to give a broad understanding of the current approaches used to manage PJIs, with a specific emphasis on the adoption and prospective improvements of the PJI-TNM categorization system, as well as an assessment of the most recent developments in local antibiotic distribution systems. Through the examination of these crucial domains, our objective is to enhance the overall comprehension and refinement of PJI therapy, with the ultimate goal of enhancing patient outcomes in this complex sector.

## Materials and methods

Study design and population

This study retrospectively observed a group of 23 patients who received therapy for septic hip or knee prostheses at the SUUB orthopedics and traumatology clinic between January 1, 2022, and February 10, 2024. The inclusion criteria consisted of patients who were diagnosed with PJI according to the criteria published by the Musculoskeletal Infection Society and the International Consensus Meeting on Orthopedic Infections.

Treatment protocols

The treatment strategy for each patient was carefully customized according to the severity and advancement of their infection. Specific techniques customized to the affected joint were utilized for surgical modifications. The surgical team used the posterolateral technique for the hip, which allowed them to have the best possible access. The knee interventions were conducted with the medial parapatellar technique, which provided sufficient visibility and control during the operation.

The recovered tissues and septic fluid from the surgeries were sent for analysis to two reputable laboratories: the laboratory at the Emergency University Hospital and the Cantacuzino Military Medical Institute in Bucharest. The purpose of these dual studies was to conduct a thorough evaluation of microbial presence and to customize antibiotic treatments with accuracy.

In most situations, a two-stage procedure was utilized. During the initial phase, a cement spacer containing antibiotics was inserted to address the infection and provide temporary joint stability. After carefully monitoring the patient and providing additional medical care following the initial surgery, the second phase of the treatment consisted of removing the temporary device and replacing it with a permanent prosthesis. In addition to the prosthesis, an antibiotic stimulant was employed to achieve elevated levels of the drug in the immediate area and reduce its presence in the overall system. The specifics of this stimulant are outlined in the results section.

When the infection was identified early in three particular cases, the levels of inflammatory markers were minimal, and the patients were in poor overall condition, it was determined that a single-stage revision was adequate. This method entailed the extraction of the contaminated prosthesis and revising it with a first-intention prosthesis while simultaneously administering specific antibiotic treatments.

PJI-TNM classification application

In our study, we employed the PJI-TNM classification system to categorize cases of prosthetic joint infections (PJI). This classification system evaluates three critical factors: the status of the tissue and implant (T), the presence and type of pathogenic microorganisms (N), and the patient's comorbidities (M). Here is a detailed description of each component used in the PJI-TNM classification.

Additionally, the classification acknowledges situations where the infection involves a previously infected implant. These cases are considered "reinfections" and are indicated by adding an "r" prefix to the classification (e.g., rT1a, rN0a).

By applying this detailed PJI-TNM classification system, we could systematically assess each case of prosthetic joint infection. This approach allowed us to tailor treatment strategies based on the severity of the tissue and implant condition, the complexity of the infection, and the overall health status of the patient, leading to more effective and individualized care [4].

Data collection and outcome measures

Data on demographic factors, surgery details, microbiological findings, and follow-up outcomes were extracted from the reviewed medical records. Uniform forms were employed to guarantee consistent collection of data, encompassing characteristics such as age, gender, kind of prosthesis, and specifics of any subsequent surgical interventions. The main outcome measures consisted of the rate at which infections were eliminated, as judged by clinical and microbiological criteria, as well as the occurrence of complications or the requirement for additional surgical procedures during the study period.

Statistical analysis

Descriptive statistics were employed to summarize the patient demographics and clinical outcomes. The Shapiro-Wilk test was used to verify the normal distribution of continuous variables. Associations between the PJI-TNM classification results and treatment outcomes were examined using Spearman's rank correlation coefficient and the chi-square test. Statistical significance was set at a p-value of less than 0.05. All analyses were performed using SPSS Statistics version 27.0 (IBM Corp. Released 2020. IBM SPSS Statistics for Windows, Version 27.0. Armonk, NY: IBM Corp.).

Ethical considerations

Before starting the study, we got ethical approval from the Ethics Council of the Emergency University Hospital Bucharest (approval number: 15678). This approval ensures that our research complies with both national and international ethical requirements for medical research. Due to the study's retrospective nature, informed consent was not required. Nevertheless, all patient data was anonymized and managed in compliance with the principles of confidentiality and privacy. The adherence to ethical principles ensured the protection of all patient-related information and the absence of any additional danger to the study participants. The study placed utmost importance on data integrity. The entirety of the gathered data was inputted into a highly secure electronic database that was specifically created for the purpose of this research. Periodic audits were performed to verify the precision and uniformity of the data entry procedure. The database was only accessible to authorized staff, and all data analyses were conducted on deidentified information to maintain the highest privacy standards.

Implementation of the PJI-TNM classification in clinical practice

In the last phase of our technique, we assessed the real-world application of the PJI-TNM classification system in regular clinical practice. This encompassed instructional workshops for healthcare practitioners at the clinic regarding the utilization of the classification system, followed by a period of surveillance to assess its incorporation into everyday clinical processes. Feedback was collected from doctors to evaluate the usefulness and effectiveness of the classification system in improving the diagnostic and therapy procedures for PJIs.

## Results

Patient demographics and infection characteristics

Between January 1, 2022, and February 10, 2024, the SUUB orthopedics and traumatology clinic treated a total of 23 patients who experienced septic advancement of their hip or knee prosthesis. The study sample consisted of 14 male and nine female participants, with ages ranging from 50 to 86 years and an average age of 68 years. These cases demonstrate a minor bias toward males in the prevalence of PJIs.

The PJI-TNM system was used to carefully examine and categorize each infection case, looking back in time. This system is a complete framework that helps in the organized assessment of the severity of the infection and the general health condition of the patient. The prevailing categorization of infections as chronic suggests that a fully developed biofilm has probably formed on the majority of prostheses, making treatment more difficult because biofilm-embedded bacteria are inherently resistant to traditional antibiotic regimens.

A number of individuals exhibited notable comorbidities, with the most prevalent ones being diabetes, obesity, and heart failure. These factors significantly affect the selection of treatment and the results, as they can worsen the patient's overall health burden and hinder recovery after surgery. Comorbidities increase the risks associated with surgical interventions, especially in controlling systemic reactions to infection and surgery.

Due to the patient's advanced age and significant risk of developing septic shock, a one-stage correction was deemed required in two specific cases. These geriatric patients were evaluated to have a poor probability of undergoing two major procedures without a high chance of death. Thus, a prompt and thorough single-step surgery was carried out to minimize the duration of their exposure to surgical stress and decrease the likelihood of serious sequelae.

All the specific features of these infections, such as the time from the original prosthesis implantation, the start of symptoms, and a thorough examination of the comorbid illnesses and how they affect the treatment process, are thoroughly recorded in Table 1. This table is an essential resource for comprehending the complex interplay between patient health profiles, infection features, and treatment outcomes.

**Table 1:** Patient demographics and infection characteristics This table details the characteristics of 23 patients treated for septic advancement of hip or knee prostheses between January 1, 2022, and February 10, 2024, including demographics, comorbidities, infection categorization using the PJI-TNM system, and specific features such as time from original implantation, symptom onset, and treatment outcomes. PJI-TNM: periprosthetic joint infection tumor, node, and metastasis, NA: not applicable

Patient ID	Age	Sex	Comorbidities	Type of intervention	PJI-TNM classification	Time since infection onset	Additional notes
1	72	M	Diabetes, obesity, smoking	Two-stage	T1a, N1a, M1	2 years	Previous urinary infection
2	65	F	Heart failure, renal failure	Two-stage	T0a, N1b, M2	18 months	N/A
3	55	M	Obesity, alcohol consumption	Two-stage	T2a, N0a, M0	6 months	Postoperative infection
4	78	F	Diabetes, heart failure, malignant tumor	One-stage	T1b, N2a, M2	3 years	High risk of septic shock
5	69	M	Diabetes, smoking, bedsores	Two-stage	T0b, N1a, M1	1 year	Chronic osteomyelitis
6	50	F	Obesity	Two-stage	T2b, N2b, M1	4 years	Previous dental infection
7	86	M	Heart failure, gastrointestinal pathologies	One-stage	T1a, N2a, M2	5 years	Patient refused further surgery
8	74	M	Heart failure, smoking	Two-stage	T1a, N1b, M1	2.5 years	Chronic pulmonary conditions
9	64	F	Obesity, diabetes	two-stage	T0a, N1a, M0	1 year	Rapid onset postoperative infection
10	59	M	Alcohol consumption, gastrointestinal pathologies	Two-stage	T2b, N2b, M2	3 years	Infection followed gastric surgery
11	82	F	Diabetes, renal failure	Two-stage	T1b, N2a, M1	4 years	Dialysis patient
12	57	M	Obesity, heart failure	Two-stage	T0b, N1a, M1	2 years	Managed heart condition
13	60	F	Smoking, alcohol consumption	Two-stage	T2a, N1b, M0	6 months	Previous lower limb infection
14	69	M	Heart failure	Two-stage	T1a, N2b, M2	5 years	Postoperative hematogenous infection
15	76	F	Diabetes, obesity	Two-stage	T0a, N0b, M1	3 years	Managed with aggressive antibiotic therapy
16	51	M	Smoking	Two-stage	T2b, N2a, M0	1 year	N/A
17	85	F	Renal failure, heart failure, diabetes	One-stage	T1b, N1a, M2	4.5 years	High surgical risk managed with care
18	61	M	Obesity, smoking	Two-stage	T0b, N2b, M1	2 years	Recurrent infection
19	53	F	Diabetes, gastrointestinal pathologies	Two-stage	T1a, N1b, M0	1.5 years	Infection post-colon surgery
20	88	M	Heart failure, obesity, smoking	Two-stage	T2a, N2c, M1	3.5 years	Infection complicated by fungal involvement
21	55	F	Heart failure, renal failure	Two-stage	T0a, N1a, M2	4 years	Severe systemic implications managed
22	63	M	Alcohol consumption, heart failure	Two-stage	T1b, N0b, M1	2 years	Late hematogenous source
23	70	F	Diabetes, obesity, heart failure	Two-stage	T2b, N2a, M1	5 years	Managed with staged revision approach

Treatment outcomes and clinical evolution

Post-treatment, the average time to achieve infection control was six months, with a range from three to 12 months. Most patients (65%) reported improved mobility and a significant increase in the range of motion. The overall condition of the patients was assessed through clinical evaluations and patient-reported outcome measures. Two patients required a one-stage revision due to the acute nature of their infections and favorable initial responses to antibiotic therapy. In contrast, the majority underwent two-stage revisions, which involved the initial placement of an antibiotic-loaded cement spacer followed by the installation of a definitive prosthesis. The outcomes indicated a high rate of infection control and satisfaction with postoperative functionality.

Highlighted clinical cases

Within the wider context of PJIs, the individual experiences of patients highlight the complex hurdles and strategic choices involved in successful care. This subchapter focuses on four particularly informative cases selected from a broader group of 23. The selection of these instances was based on their intricate nature and the crucial decision-making involved in their treatment procedures. Out of these, two cases include knee arthroplasties that are infected, and two cases involve hip arthroplasties. The complexity and severity of these infections provide a distinct chance to enhance our comprehension and approach to managing PJI.

Three of these patients necessitated a one-time revision surgery, a procedure that is not commonly employed due to its elevated dangers and strict requirements. This technique was selected due to the presence of the patients' intricate medical backgrounds, which made the decision-making process exceptionally difficult. This conversation intends to explore both the usual and unusual ways in which PJI is presented, the tactics used to intervene, the difficulties faced, and the significant lessons learned from these encounters. Every example serves as evidence of the intricate and frequently unforeseeable aspects of handling severe prosthesis infections. Together, they contribute to the ongoing discussion on the most effective approach to tackling these formidable healthcare difficulties.

Case 1

**Figure 1:**
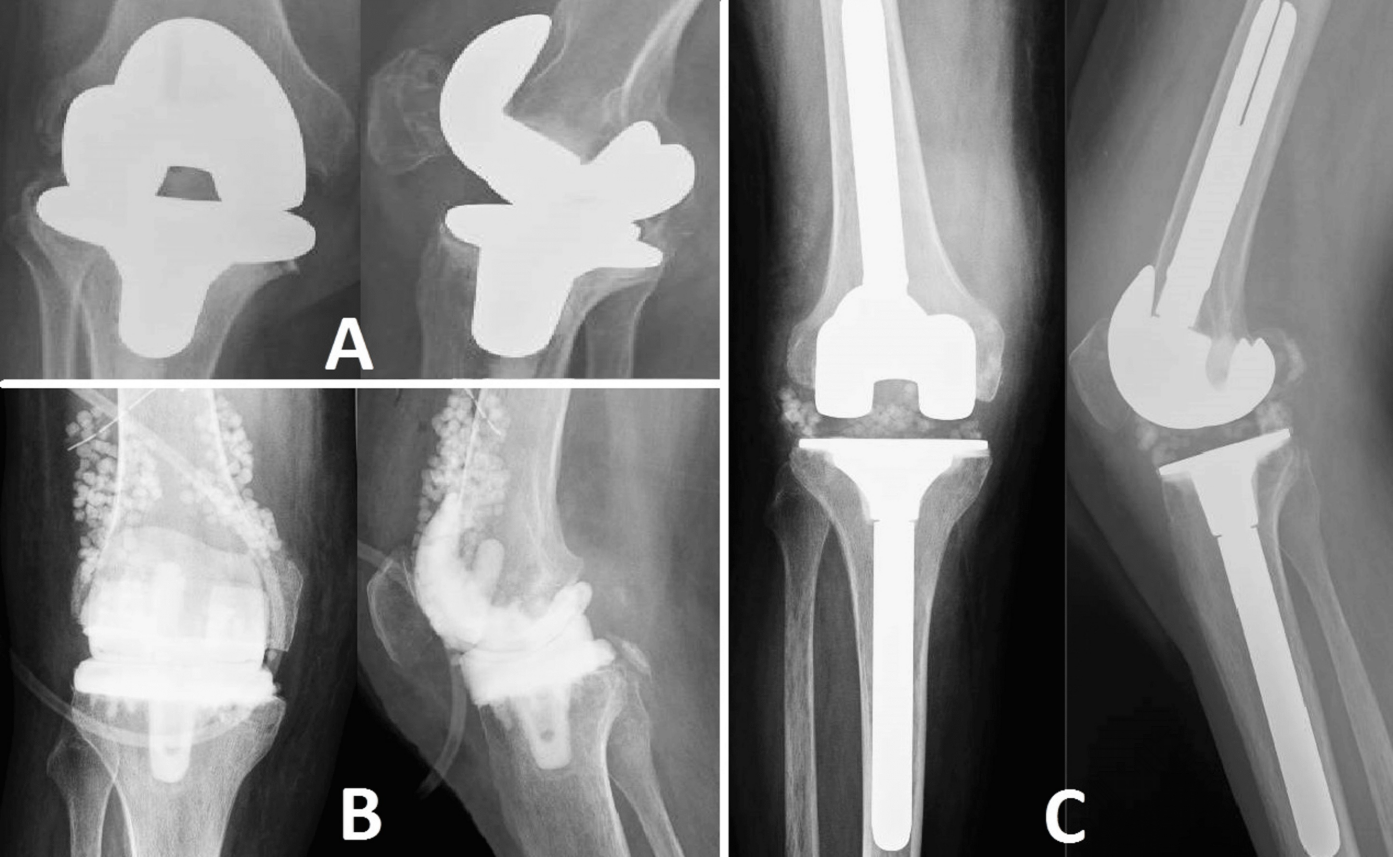
Case 1 Image A depicts a knee prosthesis exhibiting septic progression, which is defined by lysis around the components. Image B shows the condition of the infected site after debridement, with an antibiotic-loaded spacer and antibiotic beads in place. Image C displays the second phase of the revision process.

A 72-year-old man with a notable medical history of diabetes mellitus, obesity, and chronic smoking had previously undergone knee arthroplasty at the age of 63. The patient experienced many urinary infections caused by *Escherichia coli* eight years after the surgery. Although the patient ignored his initial symptoms, which made his clinical management more complicated, a proactive intervention strategy was implemented. The infected prosthesis was treated by surgical debridement of the infection focus and inserting a spacer containing antibiotics (Figure 1). Additionally, antibiotic pearls were used to give high concentrations of antimicrobial agents directly. The goal was to control the infection and minimize the harmful effects on the entire body. Following a six-month duration in which the knee infection displayed indications of significant reduction as confirmed by clinical and laboratory indicators, a revision arthroplasty was performed. This example emphasizes the difficulties of controlling PJI in patients with various comorbidities and illustrates the significance of customized interventional techniques that target both the localized and systemic elements of the infection. The effective clearance of the infection after the revision procedure provides significant knowledge about the multidisciplinary approach needed for similar high-risk instances of PJI.

Case 2

An octogenarian patient, aged 86, who underwent knee arthroplasty 12 years ago has a chronic history of cardiac problems, including significant heart failure with numerous valvulopaties, which has required the use of an oxygen device for the previous four years. The patient got a gastrointestinal infection caused by *Escherichia coli* two years ago. The infection spread to the prosthetic knee because treatment was either neglected or not given for long enough periods of time. The patient was later admitted to an advanced septic condition, with the knee showing indications indicative of a high likelihood of developing septic shock.

**Figure 2:**
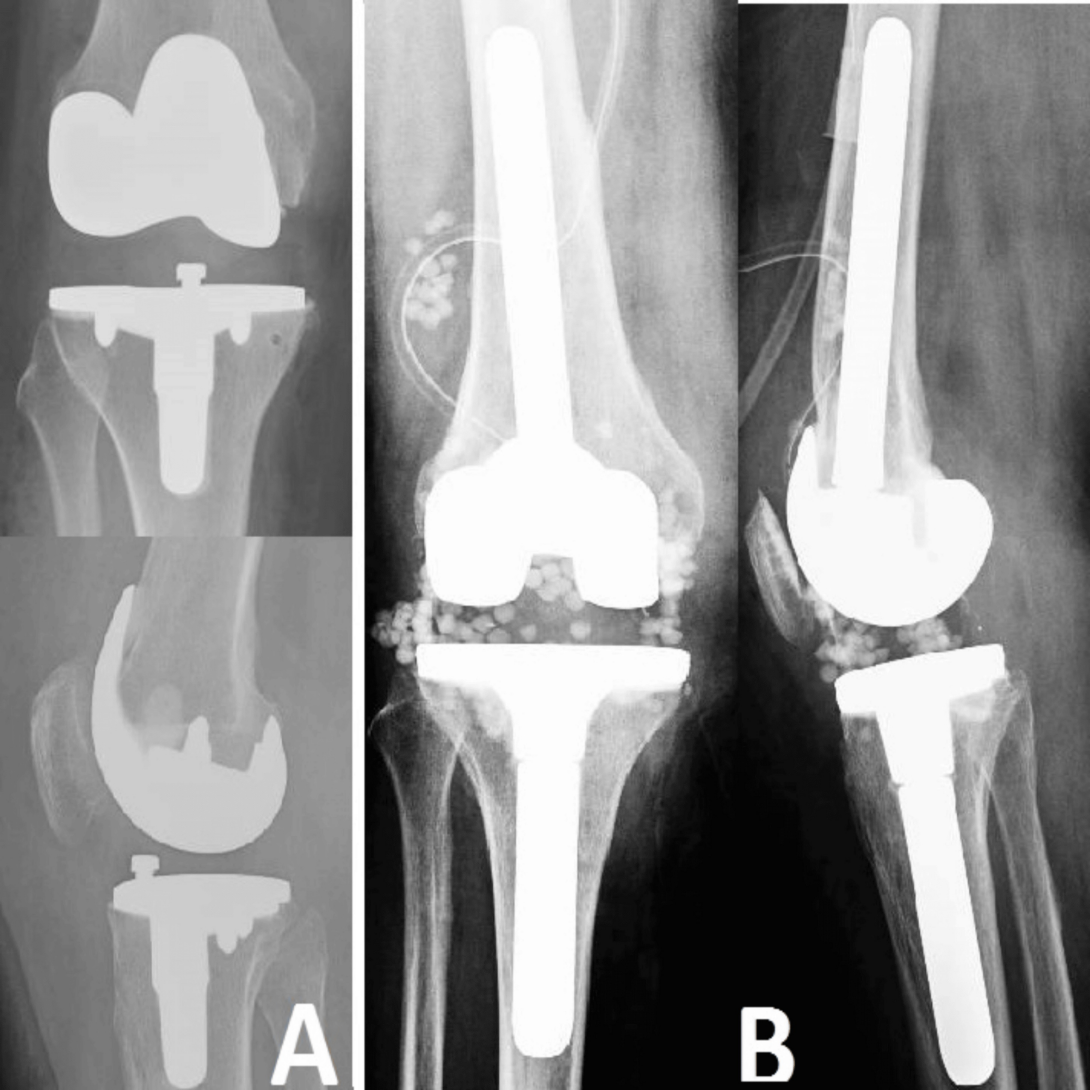
Case 2 In image A, we observe the septic progression of the knee prosthesis, characterized by peri-implant lysis. Image B shows the revision of the septic prosthesis and the antibiotic-impregnated granules.

Due to the patient's elderly status, numerous underlying health conditions, and his refusal to endure a two-stage revision process, it was determined that an immediate surgical procedure was necessary. The source of the infection was surgically removed, and a replacement knee prosthesis was implanted. Additionally, calcium pearls containing antibiotics were inserted to effectively control and reduce any infection (Figure 2). Following the intervention, there have been no documented instances of reinfection, highlighting the efficacy of the prompt and assertive treatment strategy employed in this patient, who has a high risk of recurrence.

Case 3

**Figure 3:**
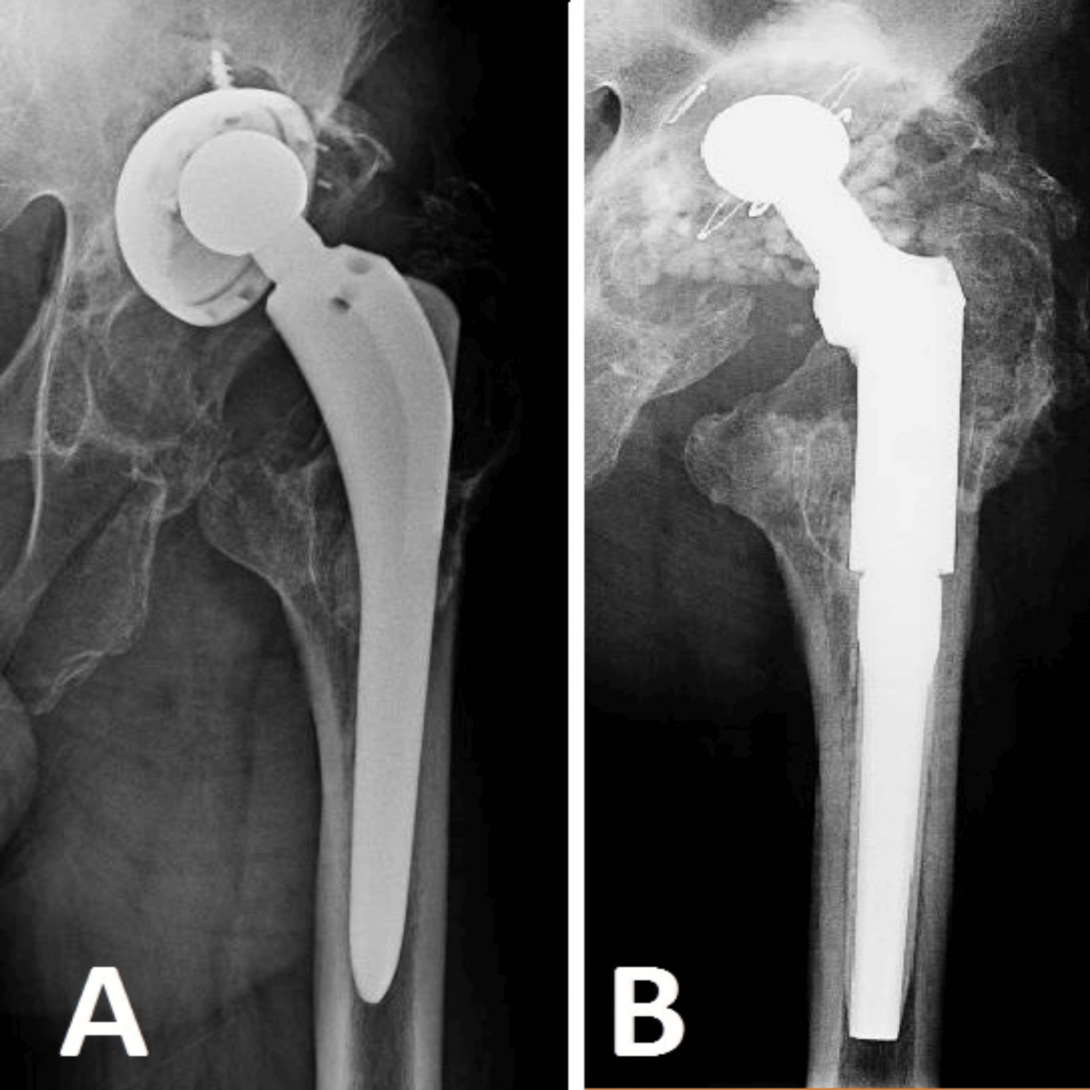
Case 3 Image A illustrates substantial devastation caused by an unaddressed persistent infection. There is clear destruction at the level of the acetabular cup, along with its mobilization. Moreover, there is notable lysis observed at the femoral level, namely in the vicinity of the trochanteric mass. Image B displays the hip revision procedure, where a revision prosthesis is used along with the incorporation of calcium granules containing antibiotics.

We present a case with a 78-year-old individual who had a total hip arthroplasty procedure a decade ago. This individual, who has diabetes and is able to consistently maintain normal blood sugar levels, is experiencing stage III NYHA heart failure. In addition, the patient has a notable medical history related to cancer, specifically breast cancer with mediastinal adenopathy and liver metastases, which was treated three years ago with mastectomy, radiotherapy, and chemotherapy. The patient is visibly undernourished and weakened, exacerbated by a lack of financial means. The patient disclosed a history of dental problems without providing specific details and mentioned sporadic utilization of oral antibiotics, which were occasionally recommended by a physician and other times self-administered, within the past three years. Approximately three years ago, she started to feel mild pain in her operated hip, which eventually became more intense. Lately, her ability to move about has been significantly limited, requiring her to use a walking frame. Following radiological evaluation (Figure 3), substantial osseous alterations are observed, attributable to a chronic infection. Further tissue cultures revealed the presence of a pathogenic infection at the hip location. Due to the patient's intricate medical history, shortened lifespan, and significant deterioration in his quality of life, it was determined that a single-stage revision of the hip arthroplasty would be undertaken. The objective of this technique was to quickly improve the patient's ability to move and prevent any further decline in their health while maximizing their functional capacity for the time they have left.

Case 4

**Figure 4:**
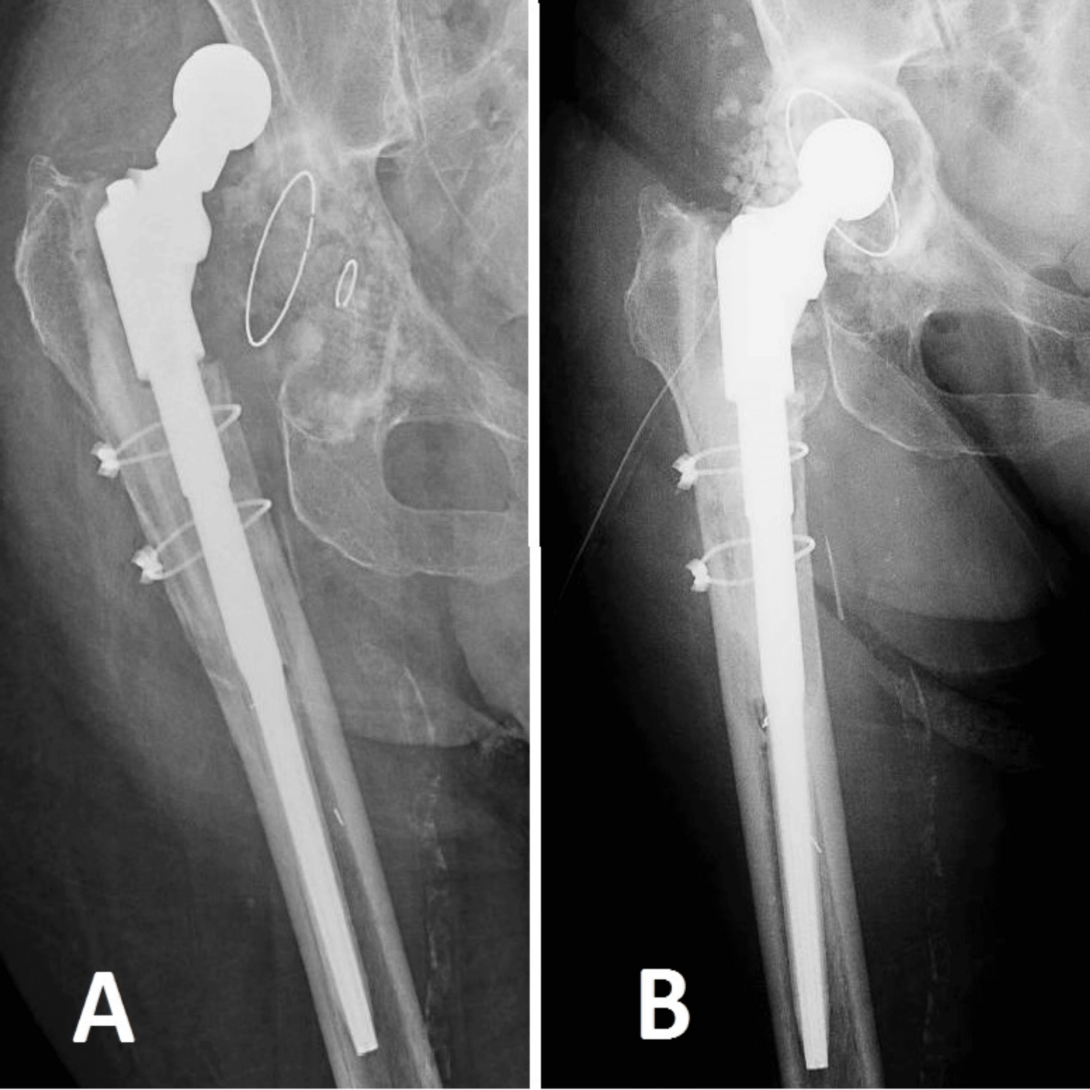
Case 4 In image A, we can observe the degradation of the acetabular cup with the dislocation of the femoral component. In Image B, we see the revision of the cup with stabilization of the hip joint.

The last case being discussed involves an 85-year-old female patient who has several coexisting medical conditions, including end-stage renal failure, which has required dialysis for the past four years, NYHA class III heart failure, and diabetes mellitus. The patient exhibits a long-lasting infection in the hip joint, resulting in substantial erosion, damage, and movement of the acetabular component (Figure 4). At first, the patient hesitated to have surgery because she had been experiencing hip problems for two years, and her other medical issues and high risk of complications made the decision more difficult. Nevertheless, the displacement of the acetabular cup has caused intense discomfort with even slight movement for a duration of almost four months, greatly impeding her ability to undergo dialysis. Given the dire circumstances, we chose to undertake a restricted intervention, focusing solely on the acetabular cup and including antibiotic-infused granules. The purpose of this method was not to fully eliminate the infectious focus but rather to offer the patient a moderately enhanced quality of life given the conditions. This case highlights the intricacies of handling severe orthopedic infections in individuals with major systemic illnesses and restricted surgical alternatives.

Laboratory findings and pathogen profiles

The laboratory investigation of infections in patients with prosthetic joints at our clinic reveals a high prevalence of Gram-positive bacteria, specifically *Staphylococcus aureus*, found in 39.1% of cases, and *Staphylococcus epidermidis* in 17.4%. Methicillin-resistant *Staphylococcus aureus* (MRSA) strains were detected in 13.0% of the cases of *Staphylococcus aureus* (Table 2). Additional Gram-positive infections comprised *Streptococcus agalactiae* and *Enterococcus faecalis*, constituting 4.3% and 13.0% of the cases, respectively. The Gram-negative pathogens, namely *Escherichia coli*, *Pseudomonas aeruginosa*, *Serratia marcescens*, and *Klebsiella pneumoniae*, individually accounted for fewer than 9% of the total cases. An antibiogram was conducted for each identified pathogen to customize the antibiotic treatment specifically based on the discovered resistance patterns, thereby enabling focused antimicrobial therapy. Furthermore, antibiotic-laden beads were tailored according to these antibiograms to provide efficient localized drug administration precisely at the site of infection. This focused approach demonstrates a deliberate change in strategy toward precision medicine in the management of prosthetic joint infections, with the goal of improving treatment effectiveness and optimizing patient results.

**Table 2:** Laboratory findings and pathogen profiles This table presents the prevalence of various pathogens in prosthetic joint infections, highlighting a predominance of Gram-positive bacteria such as *Staphylococcus aureus* and *Staphylococcus epidermidis*, and details the tailored antibiotic treatments based on specific resistance patterns, including the use of antibiotic-laden beads for localized therapy. MRSA: methicillin-resistant *Staphylococcus aureus*

Microorganism	No. of cases	Percentage (%)
Gram-positive bacteria		
*Staphylococcus aureus* (total)	9	39.1%
MRSA	3	13.0%
Staphylococcus epidermidis	4	17.4%
Streptococcus agalactiae	1	4.3%
Enterococcus faecalis	3	13.0%
Gram-negative bacteria		
Escherichia coli	2	8.7%
Pseudomonas aeruginosa	1	4.3%
Serratia marcescens	1	4.3%
Klebsiella pneumoniae	1	4.3%

## Discussion

The most important findings of the study were that PJIs continue to be a crucial obstacle in orthopedic surgery, marked by substantial diversity in how they appear and the results they yield. The intricacy of these infections necessitates a classification system that goes beyond the conventional binary categorization of acute or chronic kinds. The implementation of the PJI-TNM categorization system signifies a noteworthy progression, reflecting the fundamental principles of the oncological TNM system. This categorization system classifies the infection according to the specific condition of the implant and the surrounding tissues (T), the specific type of microorganisms causing the infection (N), and the other medical conditions the patient may have (M). Employing a nuanced approach enables the development of a customized therapy strategy, which has the potential to enhance clinical results by effectively addressing the individual needs and risks of each patient [2,14-16].

Osteomyelitis has similar diagnostic and therapeutic difficulties as PJIs, particularly in regard to the methods used for debridement and administration of antibiotics. To effectively manage the infection, it is typically necessary to remove necrotic bone and utilize antibiotic carriers. PMMA and calcium sulfate (Stimulan) are crucial carriers among these options. PMMA, a non-biodegradable alternative, has been widely utilized to administer concentrated doses of antibiotics to specific areas [10,17]. Nevertheless, surgical extraction becomes necessary after the completion of therapy, which can potentially complicate the treatment procedure. On the other hand, biodegradable alternatives such as calcium sulfate have the benefit of being able to be absorbed by the body, which means there is no need for removal and also lowers the chance of requiring a second surgery [10,18].

Biomaterials have had a profound effect on the treatment of bone infections. Advancements in antibiotic-loaded bone cement and other carriers have made it possible to administer drugs directly to the site of infection. This targeted strategy reduces the occurrence of widespread adverse effects and improves the effectiveness of antibiotics against biofilms, which pose a significant challenge in the treatment of PJIs due to their resistance to conventional therapies. Research has demonstrated that the combination of various antibiotics can enhance the release patterns and effectiveness against bacteria, which is essential for the treatment of infections related to orthopedic implants (Table 3) [1,19].

**Table 3:** Studies DAIR: debridement and implant retention, CS: calcium sulfate, HO: heterotrophic ossification

Study	Sample size	Joints affected	Treatment	Antibiotic used	Follow-up period	Complications observed	Infection recurrence rate
McPherson et al. (2013) [24]	250 joints	142 knees, 108 hips	Aseptic revision, DAIR, revision	Vancomycin, tobramycin	3, 6, 12 months	Wound drainage, hypercalcemia, HO	6 cases (3 knees, 3 hips)
Ferguson et al. (2014) [25]	161 cases	88 knees, 73 hips	Biodegradable CS carrier	Tobramycin	-	-	11 cases (6.8%)
Flierl et al. (2017) [26]	33 joints	6 hips, 27 knees	DAIR, antibiotic-loaded CS beads	Vancomycin, tobramycin	3-30 months (avg 12.7)	Persistent drainage, high recurrence	16 of 33 cases (48%)
Ferrando et al. (2017) [27]	12 patients	13 knees, 2 hips	Cavitary bone defect treatment	Vancomycin, gentamicin	-	-	1 case (12.5%)
Kallala et al. (2018) [28]	755 joints	456 knees, 299 hips	Various revisions (infection, DAIR)	Vancomycin, tobramycin	6 weeks, 3, 6, 12 months, bi-annually	Wound drainage, hypercalcemia, HO	Not reported
Zhou et al. (2020) [29]	42 patients	42 knees	Single-stage treatment, local debridement	Vancomycin, gentamicin	-	Aseptic drainage, pain, discomfort, claudication	5 of 42 cases (11.9%)
McNally et al. (2016) [30]	62 cases	38 knees, 24 hips	Single-stage treatment, biocomposite	Gentamicin	-	Sterile wound drainage, amputation, nonunion	2 cases (3.2%)
Kallala et al. (2015) [19]	15 joints	6 knees, 9 hips	Revision for infected arthroplasty	Vancomycin, gentamicin	6 weeks, 3, 6, 12 months	Wound discharge, hypercalcemia, HO	1 case

Moreover, there is an ongoing discussion regarding the optimal strategies for handling PJIs and osteomyelitis, specifically regarding the timing of interventions and the selection between surgical and conservative therapy. The categorization of infection stages, as outlined in classifications such as the PJI-TNM, aids in determining whether prompt and intensive therapy or a more cautious strategy with gradual interventions is suitable. The timing of surgical intervention is a critical factor that can greatly impact the outcome, particularly in acute instances where early intervention can avoid the formation of a biofilm [7,12,20].

The utilization of biodegradable antibiotic transporters, such as calcium sulfate, within the specific context of our research population exhibited notable advantages. These carriers were especially beneficial for patients with severe comorbidities or those at a high risk of surgical complications, as they decreased the necessity for additional procedures to remove the antibiotic delivery system. The targeted use of potent antibiotics efficiently controlled infections while considering the specific health limitations of each patient. In cases where a two-stage revision was required, the temporary use of antibiotic-loaded spacers not only offered mechanical stability but also played a significant role in controlling infections. This was essential in preparing these patients for the insertion of a permanent prosthesis later on [15,21-23].

Moreover, our study's approach to microbial diagnostics and tailored antibiotic therapy based on specific pathogen profiles highlighted in the laboratory findings emphasize the importance of precision medicine in PJI management. By identifying the exact bacterial strains and their antibiotic sensitivities, treatment could be customized, which is crucial in dealing with infections complicated by resistant or unusual pathogens [1,2,16,29].

The case-based approach in our study also highlighted several key learnings. For example, one patient, previously managed for a chronic urinary infection with Escherichia coli, later developed a PJI, illustrating the route and implications of hematogenous spread. Another patient, a diabetic with a history of cancer treatment, presented unique challenges due to compromised physiological resilience and a complex infection history. These cases illustrate the critical need for an integrated approach that considers the full spectrum of patient health beyond the localized site of infection.

Furthermore, it is imperative to investigate and tackle the socio-economic variables that impact the management of PJIs. The economic impact of these infections on healthcare systems is substantial, stemming from both the expenses associated with treatments and the additional demands for extended hospitalization and heightened postoperative care. Health policymakers should prioritize allocating funds for research on cost-effective treatment methods and providing support for technology that can lower the overall financial burden of PJIs [8,14,27].

## Conclusions

Although the therapy of PJIs has made tremendous progress, there is still a discrepancy between the development of new medicines and their use in clinical practice. To bridge this gap, a collaborative endeavor is necessary among healthcare professionals, researchers, and policymakers to guarantee that all patients may reap the advantages of these advancements. By creating a conducive atmosphere that encourages the swift implementation of sophisticated categorizations, customized therapies, and state-of-the-art diagnostic techniques, the medical field can improve patient results and mitigate the effects of PJIs on both individuals and healthcare systems.
